# ATF4 Contributes to Abdominal Aortic Aneurysm Formation *via* Modulating M1 Macrophage Polarization and Inflammation

**DOI:** 10.14336/AD.2024.0116

**Published:** 2025-06-10

**Authors:** Huahui Yu, Xiaolu Jiao, Qianwen Lv, Linyi Li, Yunhui Du, Chaowei Hu, Zhiyong Du, Fan Li, Yu Wang, Xiaoqian Gao, Lijie Han, Xuechun Sun, Dong Chen, Yanwen Qin

**Affiliations:** ^1^The Key Laboratory of Remodeling-Related Cardiovascular Diseases, Beijing Anzhen Hospital, Capital Medical University, Ministry of Education, Beijing Institute of Heart Lung and Blood Vessel Disease, Beijing, China.; ^2^Department of Pathology, Beijing AnZhen Hospital, Capital Medical University, Beijing, China

**Keywords:** abdominal aortic aneurysm, activating transcription factor 4, inflammation, macrophage polarization

## Abstract

Abdominal aortic aneurysm (AAA) is a potentially life-threatening vascular disease primarily in the male elderly population, but there is a lack of approved medical therapies to prevent the progression and rupture of AAA. Activating Transcription Factor 4 (ATF4) has been established to be involved in cardiovascular diseases, such as heart failure and calcific aortic valve disease. However, the role of ATF4 in the pathogenesis of AAA remains unclear. We found that ATF4 expression was significantly increased in patients with AAA and mouse models of AAA and was mainly confined to macrophages in arteries. ATF4 knockdown significantly attenuated aneurysm formation in experimental mouse model of AAA, while ATF4 overexpression promoted the development of AAA. RNA sequencing suggested that ATF4 was strongly related to the biological function of acute inflammatory response. Macrophages-specific ATF4 knockout significantly reduced the incidence and development of AAA, and decreased M1 polarization of macrophages in mice. Sphingomyelin phosphodiesterase 3 (SMPD3), a regulator of inflammatory responses in monocytes/macrophages, has been identified as a target gene of ATF4 through RNA sequencing, ChIP sequencing, and standard ChIP analyses. ATF4 induces M1 polarization of macrophages through the activation of SMPD3, thereby promoting inflammatory responses. Together, these results suggest that ATF4 mediated macrophage M1 polarization by regulating the expression of target genes SMPD3, leading to an increased inflammatory response, which further promotes the formation and development of AAA. These findings suggest ATF4 may be a new therapeutic target for AAA.

## INTRODUCTION

Abdominal aortic aneurysm (AAA) is characterized by localized dilation of the aortic diameter, measuring ≥3 cm or surpassing the normal vascular diameter by more than 50%. Once ruptured, the mortality rate for AAA significantly is up to more than 90% [1]. AAA usually occurs in the male elderly population and is associated with risk factors such as hypertension, smoking, atherosclerosis, and other vascular diseases [2, 3]. Currently, endovascular aneurysm repair and open surgical repair are the main treatment for AAA, but effective pharmacological therapies to inhibit AAA expansion and prevent aneurysm rupture have not been established [4]. This is partially due to the incomplete understanding of the pathophysiologic mechanisms underlying the occurrence and development of AAA.

The main pathophysiological processes contributing to the formation of AAA include chronic inflammatory infiltration, smooth muscle cell apoptosis, oxidative stress, extracellular matrix degradation, and the upregulation of proteolytic pathways [5]. These factors act synergistically, leading to the persistent expansion of the abdominal aorta. Inflammation is the initiator of the formation of AAA and is involved in the entire progression of the AAA. Studies have shown that inflammation is the initiator of the formation and is involved through the whole progression of AAA. The roles of inflammation mediated by macrophages in the formation and progression of AAA up to rupture has been widely documented [6]. Macrophages are classified into two subsets as classically activated macrophages (M1-like) and alternatively activated macrophages (M2-like), which play a crucial role in resolving the initial inflammatory phase. Typically, M1 cells exert a proinflammatory effect and promote matrix degeneration, whereas M2 cells contribute to the resolution of inflammation and alleviate tissue remodeling [7]. Emerging evidence has indicated that the regulation of differently polarized macrophages in situ may be a potential approach to modulate inflammation and AAA formation [8]. Macrophage polarization plays an important role in the progression of AAA, and would provide a potent therapeutic target for AAA treatment [9]. Although macrophage polarization has been involved in the progression of AAA, the mechanisms of macrophage polarization have not been clearly defined.

Activating transcription factor 4 (ATF4), a key factor in the unfolded protein response and the integrated stress response, belongs to the basic leucine zipper transcription factor family. As previously reported, ATF4 is a stress-inducible gene that is upregulated in a variety of diseases, including cardiac, pulmonary, and neoplastic diseases [10-12], as well as acute vascular injury [13]. ATF4 has been reported to be associated with cardiovascular disease. ATF4 plays a critical role in the heart failure under conditions of hemodynamic stress by regulating the cytosolic and mitochondrial production of NADPH [10]. ATF4 plays a multifaceted role in the development of pathologies where ATF4 controls many cellular signaling pathways, such as autophagy, oxidative stress, and inflammatory response [14]. However, the function of ATF4 in AAA remains largely uncharacterized. We hypothesized that ATF4 may participate in AAA.

In the current study, we demonstrated the effect of ATF4 on the pathogenesis of AAA and explored its underlying molecular mechanisms using human pathological specimens, mice experimental AAA models and cellular models. Based on the experimental data, we identified the novel mechanism of ATF4-mediated macrophage polarization and inflammation in the pathogenesis of AAA development, which would provide a potential therapeutic target for the prevention and treatment of AAA.

## MATERIALS AND METHODS

### Human Aortic Tissue Samples

Aortic aneurysm surgical specimens were obtained from patients with AAA undergoing open surgical repair at Beijing Anzhen Hospital, Capital Medical University. Adjacent non-aneurysmal aortic segments were excised from the same patients as controls. According to the 2018 Society of Vascular Surgery Practice Guidelines, patients with low surgical risk or acceptable fusiform AAA ≥5.5 cm accepted elective repair. All experiments using human samples in this study were approved by the Medical Ethics Committee of the Beijing Anzhen Hospital, Capital Medical University, and were conducted according to the Declaration of Helsinki principles. All participants gave written informed consent [15]. Human specimens were fixed in 10% formalin, embedded in paraffin, and sectioned at 5-μm thickness (n=6 per group).

### Animal Model

Animal experiments were conducted following approval from the Ethics Committee of Capital Medical University and strictly adhering to the guidelines in the National Institutes of Health Guide for the Care and Use of Laboratory Animals. All experiments involving animals were carried out in the SPF Animal Laboratory of Beijing Anzhen Hospital. All mice used in the experiments were bred under specific pathogen-free conditions at the animal facilities of Beijing Anzhen Hospital. The mice were housed under standard conditions with a 12-hour light and 12-hour dark cycle.

In this study, only male mice were used because of the lower incidence of AAA in female mice compared to male mice, as reported in several mouse models [16]. Male ApoE-/- mice were purchased from Beijing Huafukang Biotechnology (Beijing, China). ATF4 flox/flox (ATF4^fl/fl^) mice (C57BL/6J background) were acquired from Cyagen Biosciences (Cyagen Biosciences, Guangzhou, China). Macrophage-specific ATF4 knockout mice (ATF4^Lyz2KO^) were generated by crossing ATF4^fl/fl^ mice with Lyz2 -Cre transgenic mice. Lyz2 -Cre mice were from the Viewsolid Biotech. Mousetail genomic DNA was amplified by PCR for genotyping. The in vivo experiment was divided into three parts.

In the first part of our study, ApoE -/-mice (male, aged 8-10 w) were randomly divided into the Saline (n = 6) group and Ang II (n = 6) group. The saline group received a continuous subcutaneous infusion of saline, whereas the Ang II group were administered a continuous subcutaneous infusion of Ang II (1000 ng/kg per min) through an osmotic pump (Alzet MODEL 1007D; DURECT, Cupertino, CA) for a period of 28 days. Mice were anesthetized by intraperitoneal injection of a 1% sodium pentobarbital, followed by the implantation of mini pumps. During the 28-day Ang II infusion, any dead mice that were autopsyied to determine if the cause of death was related to aneurysm complications. AAA tissue and healthy aorta tissue were collected to determine the expression of ATF4 and subsequent RNA-seq analysis.

In the second part of the study, to explore the role of ATF4 during the occurrence and development of AAAs. ATF4-knockdown mice were generated by injection with an adeno-associated virus serotype-9 (AAV-9) encoding a short hairpin RNA (shRNA) specifically targeting mouse ATF4. ATF4-overexpression mice were generated by injection with an AAV-9 encoding recombinant ATF4. The target sequence of mouse ATF4 shRNA was as follows: 5‘-GCTGCTTACATTACTCTAATC-3’. AAV9 carrying this sequence (AAV-sh-ATF4), or the scramble control (AAV-control) was generated by Shanghai Genechem Co. Ltd. The mouse ATF4 complementary DNA (cDNA) (NM_009716.3) was cloned into the pAAV-C-FLAG-WPRE plasmid. AAV9 carrying the plasmid (AAV-ATF4) or the scramble control (AAV-control) was also generated by the Shanghai Genechem Co. Ltd. The recombinant AAV vectors were injected intravenously into mice via the mouse tail vein (2.14 × 10^11^ vg/body).

Two weeks after AAV transfection, ApoE^-/-^ male mice (8 weeks of age) were divided into four groups: Saline+AAV-control (n = 6), AngII+AAV-control (n = 20), AngII+AAV-sh-ATF4 (AngII+sh-ATF4, n = 15), and AngII+AAV-ATF4 (AngII+oe-ATF4, n = 15). As previously described, mice in all groups were injected with Saline or AngII pumps for 28 days.

In the third part of the study, 8-week-old male ATF4^Lyz2KO^ mice and littermate controls (ATF4^fl/fl^) were used for the experiments. Mice divided into four groups: ATF4^fl/fl^ +Saline (n=6), ATF4^Lyz2KO^ +Saline (n=6), ATF4^fl/fl^ +AngII (n=15) and ATF4^Lyz2KO^+AngII (n=15). AngII-induced AAA facilitated by transduction of an AAV (sero-type 8) carrying a gain-of-function mutation of the mouse PCSK9 (AAV-mPCSK9 D377Y, GeneChem) in mice was performed following previous studies [17]. In brief, mice were intravenously injected with 2 × 10^11^ vector genomes of AAV-mPCSK9 D377Y and transitioned to a high-fat, high-cholesterol (HFHC) diet (Research Diets, D12108C) to induce hypercholesterolemia. Two weeks after AAV injection, the mice underwent subcutaneous injection of AngII and continued on the HFHC diet for an additional 4 weeks.

All mice were executed after intraperitoneal injection of 1% sodium pentobarbital, and their aortic tissues and plasm were collected for further analysis.

### Enzyme-linked immunosorbent assay (ELISA)

The levels of mouse interleukin-1 (IL-1β), IL-6, and Tumor Necrosis Factor α (TNF-α) were measured via ELISA kits (RX203063M, RX203049M, RX202412M, Ruixin BIO, China). The tests were carried out according to the instructions for product use.

### Measurement of Systolic Blood Pressure in Mice

Systolic blood pressure in awake mice was monitored weekly using a noninvasive tail-cuff system (BP-98A; Softron, Tokyo, Japan), starting 1 w before AngII infusion and continuing throughout the study. The measurements were performed once a week from 9 to 11 am in a quiet room. A preliminary cycle of 10 cycles was performed to allow the mice to adapt to the inflatable cuff before measurements were started. Three consecutive blood pressure measurements were taken in each mouse and averaged.

### Quantification of AAA

Mice were anesthetized with 1% isoflurane and scanned by a high-resolution micro-ultrasound system (Vevo2100; Toronto, Canada) using M-mode with a 30 MHz sensor. The diameter of the artery was measured by color Doppler examination as described previously [18]. Abdominal aortic aneurysm is defined as an increase in diameter to 1.5 times that of the normal aorta. In addition, in the AAA mouse model, any stripping resulting in intramural haematoma (even if the actual dilatation is only 1.1 times that of the normal aorta) should be taken into account [19].

### Histopathology and immunohistochemistry

Tissues were cut into 5-μm sections on a microtome. All sections were numbered consecutively and the same number of sections from each group was subjected to staining.

For immunohistochemical staining, tissue sections were deparaffinized and rehydrated by successive washes of xylene, 100% ethanol, 95% ethanol, 80% ethanol, and water. After rehydration, the sections were permeabilized with 0.01% Triton X-100 in phosphate-buffered saline (PBS), blocked with 10% goat serum, and then incubated with primary antibodies at 4°C overnight. The next day, sections were incubated with the appropriate horseradish peroxidase-conjugated secondary antibody and counterstained with hematoxylin. The following primary antibodies were used: rabbit anti-ATF4 (1:200, CST), rabbit anti-IL-6 (1:200, Abcam), rabbit anti-IL-1β (1:200, Proteintech), mouse anti-MPC-1 (1:200, Proteintech), rat anti-F4/80 (1:200, Abcam), mouse anti-MMP-9 (1:200, Abcam), rabbit anti-iNOS (1:200, Proteintech) and rabbit anti-CD38 (1:200, Proteintech). For all immune-histochemistry studies, normal rabbit IgG was used as the negative control.

Images were obtained with a Ni-U Nikon Upright Microscope (Nikon, Tokyo, Japan). At least six images per section were statistically analyzed using ImageJ software. Relative expression was calculated as a percentage of total pixel intensity of each image.

### In situ hybridization

*In situ* hybridization (ISH) was performed using digoxigenin (DIG)-labeled probes as previously described. Briefly, tissue sections of 5-μm thickness were incubated with digoxigenin-labeled RNA probes in hybridization buffer overnight. The sections were then incubated with an alkaline phosphatase-conjugated anti-digoxigenin antibody (Roche) and developed with nitro blue tetrazolium/bromochloroindolyl phosphate (NBT/BCIP) as substrates. Slides were counterstained with hematoxylin. The ATF4 probe were described previously [20]. Images were obtained with a Ni-U Nikon Upright Microscope (Nikon, Tokyo, Japan). For statistics, at least six images per section were analyzed using ImageJ software. Relative expression was calculated as a percentage of total pixel intensity of each image.

### Double Immunofluorescence Staining

Tissue sections were antigenically recovered and blocked as described above, followed by incubation with rabbit anti-ATF4 antibody (1:200; CST) together with rat anti-F4/80 (1:200, Abcam), mouse anti-α-SMA (1:200, ZSGB-BIO) or mouse CD31 (1:200, Santa Cruz) antibodies in PBS containing 0.2% Triton X-100 at 4°C overnight. After three washes using the same buffer, the sections were then incubated with Alexa Fluor 555-conjugated rat antibodies and Alexa Fluor 488-conjugated antigenically recovered and blocked antibodies aga for 2 hours at room temperature. Following the incubation period, the sections were washed, and fluorescent images were taken using a Nikon TS2-S-SM microscope equipped with a Nikon DS-Qi2 camera.

### Western blot

Tissues and cells were homogenized in an ice-cold suspension buffer supplemented with a proteinase inhibitor cocktail (Sigma-Aldrich). Protein extraction was carried out using a kit containing protease inhibitors and a protein phosphatase inhibitor cocktail. The protein concentrations were determined using a BCA Protein Assay Kit (Thermo Fisher Scientific, Waltham, MA). Subsequently, equal samples were loaded, separated, and transferred to a membrane (Pierce; Thermo Fisher Scientific). Following membrane transfer, the membranes were blocked, incubated overnight at 4°C with the primary antibodies: rabbit anti-ATF4 antibody (1:1000, CST), rabbit anti-SMPD3 antibody (1:1000, Santa), mouse anti-MMP-9 (1:1000, Proteintech), rabbit anti-iNOS (1:1000, Proteintech) or rabbit anti-CD38 (1:1000, Proteintech). GAPDH was the internal reference (1:1000; CST). Then, the membranes were washed with Tris-buffered saline containing Tween 20, and then incubated with secondary antibodies (1:10000 dilution; CST) for 1 hour at room temperature. Blots were then washed, incubated with SuperSignal™ WestFemto Maximum Sensitivity Substrate (Thermo Fisher Scientific), and analyzed using a ChemiDoc™ Touch Imaging System (Bio-Rad, Hercules, CA). The relative expression was normalized to GAPDH expression.

### In situ zymography

Fresh abdominal aortic cryosections embedded in OCT were subjected to analysis through in situ zymography employing the MMP fluorogenic substrate DQ-gelatin-FITC (Invitrogen). The cryosections were incubated with 40 μg/ml of the quenched fluorogenic substrate DQ-gelatin-FITC in PBS for 1 hour at 37 °C. The fluorogenic substrate, with a fluorescent tag, remained in a caged state (showing no fluorescence) until cleaved by gelatinase. After incubation, excess fluorogenic substrate was washed away with PBS, and the sections were photographed using a Nikon TS2-S-SM microscope. Quantitative analysis of fluorescence intensity was conducted using ImageJ software.

### In situ dihydroethidium (DHE) staining

In situ dihydroethidium (DHE) staining (Beyotime, #S0063) was conducted to evaluate superoxide production following the manufacturer’s protocol. The aortic sections, freshly frozen and 6μm thick, were incubated with DHE dye, resulting in ethidium staining (appearing red). Subsequently, photos were captured using a fluorescence microscope.

### RNA-sequencing and data analysis

ApoE^-/-^ mice were infused with Saline or Ang II for 28 days. Aortas were collected for RNA extraction. The total RNA of mouse aorta tissue was extracted by TRIzol method (Invitrogen). The mRNA was reverse transcribed into double strand cDNA fragments. Library construction was performed by Illumina's TruSeq RNA Library Prep Kit per manufacturer's protocol (Illumina, San Diego, CA). Final library quality control was performed by DNA High Sensitivity Qubit Kit (Invitrogen), the Bioanalyzer High Sensitivity Chip Kit (Agilent), and the qPCR machine (Agilent). qPCR was performed via Illumina Universal Library Quantification Kit (KAPA Biosystems). RNA libraries were then sequenced at 10pM on the Illumina HiSeq 2000 to generate 100 bp pair-end reads. After adaptor-removing and quality control filtering, the clean reads were mapped to mouse genome (Mm9) using Tophat2 (v2.0.9). Cufflink (v2.0.0) was used to identify differentially expressed genes (DEGs) between samples. Significant DEGs of each pairwise comparison was selected (p value<0.05). Pathway analysis, predominantly based on the Kyoto Encyclopedia of Genes and Genomes (KEGG) database, was employed to determine the significant functions and pathways of DEGs.

### Cell Culture conditions and treatments

RAW 264.7 cells were obtained from the Shanghai Xinyu Biotech co., Ltd. RAW 264.7 were cultured in the DMEM medium (Science cell, CA) supplemented with 10% fetal bovine serum (FBS) and 1% penicillin/streptomycin solution in a humidified atmosphere containing 5%CO_2_/95% air at 37ºC. RAW264.7 cells were treated with TNF-α (10 ng/ml) for 24 h in the TNF-α stimulation study. RAW264.7 cells were treated with LPS (100 ng/ml) for 24 h to induce M1 polarization. SMPD3 inhibitor GW4869 (Sigma D1692, 10 µM/ml) treatment 24h was to inhibit SMPD3 in RAW264.7 cells.

### Cell transfection

We used a lentiviral ATF4 specific shRNA, ATF4-overexpression vector and SMPD3 shRNA (Shanghai Genechem) to transfect RAW264.7 cells. A Stealth Negative Control Lentiviral Vector (Shanghai Genechem) was used as a control (GFP). Cells were infected with 100 MOI Vector for 48 hours and then cultured in the presence of suramin medium for 72 hours before experimental assays. Stable transfections of these vectors were performed according to the manufacturer’s instructions. Stably transformed cell lines were screened and maintained by puromycin (5 μg/mL). Stable RAW264.7 strain was used in subsequent experiments.

### Chromatin immunoprecipitation sequencing (ChIP-seq)

RAW264.7 cells were transfected with GFP or ATF4-overexpression vector, cross-linked with 1% formaldehyde and performed overnight with or without ATF4 antibody (11815, CST). Acquired DNA samples prepared for Sequencing Library using TruSeq Nano DNA Sample Prep Kit (Illumina, San Diego, CA, USA). Sequencing was performed on Illumina HiSeq 4000 platform. All experiments were performed with manufacturer’s instructions. Peaks with a p value ≤0.01 were accepted for further analysis. FC ≥ 2.0 and p value ≤ 0.001 were used to classify differentially enriched regions. Recovered DNA was analyzed by qPCR. Primers spanning the promoter regions of Sphingomyelin phosphodiesterase 3(SMPD3) gene were used to amplify input and immunoprecipitated DNA (F:5’ TGGGTTTT GTCGTATGGTGAA 3’, R:5’ AAACCTCTCATCCC AGAACAGTC 3’). All samples were performed in at least triplicates from at least 2 independent experiments, and data were normalized to percent input.

### Representative images

Choose high-quality, clear images with clean backgrounds and obvious features, which can fully represent the features of the remaining images.

### Statistical analysis

Data were analyzed using Prism 8.0 software (GraphPad Software, San Diego, CA) and are presented as mean ± SD. Each experiment was repeated at least three times. All of the data passed normality and equal variance tests. Differences between 2 groups were compared using unpaired Student’s t-test. Differences among 3 or more groups were compared using 1-way analysis of variance (ANOVA), followed by Tukey’s post hoc test for multiple pairwise comparisons. The Kaplan-Meier survival curve was used to analyze the survival percentages of saline- or Ang II-infused mice. A P value lower than 0.05 was considered statistically significant (*).

## RESULTS

### ATF4 expression was significantly increased in human and mouse AAA tissues

**Figure 1.**
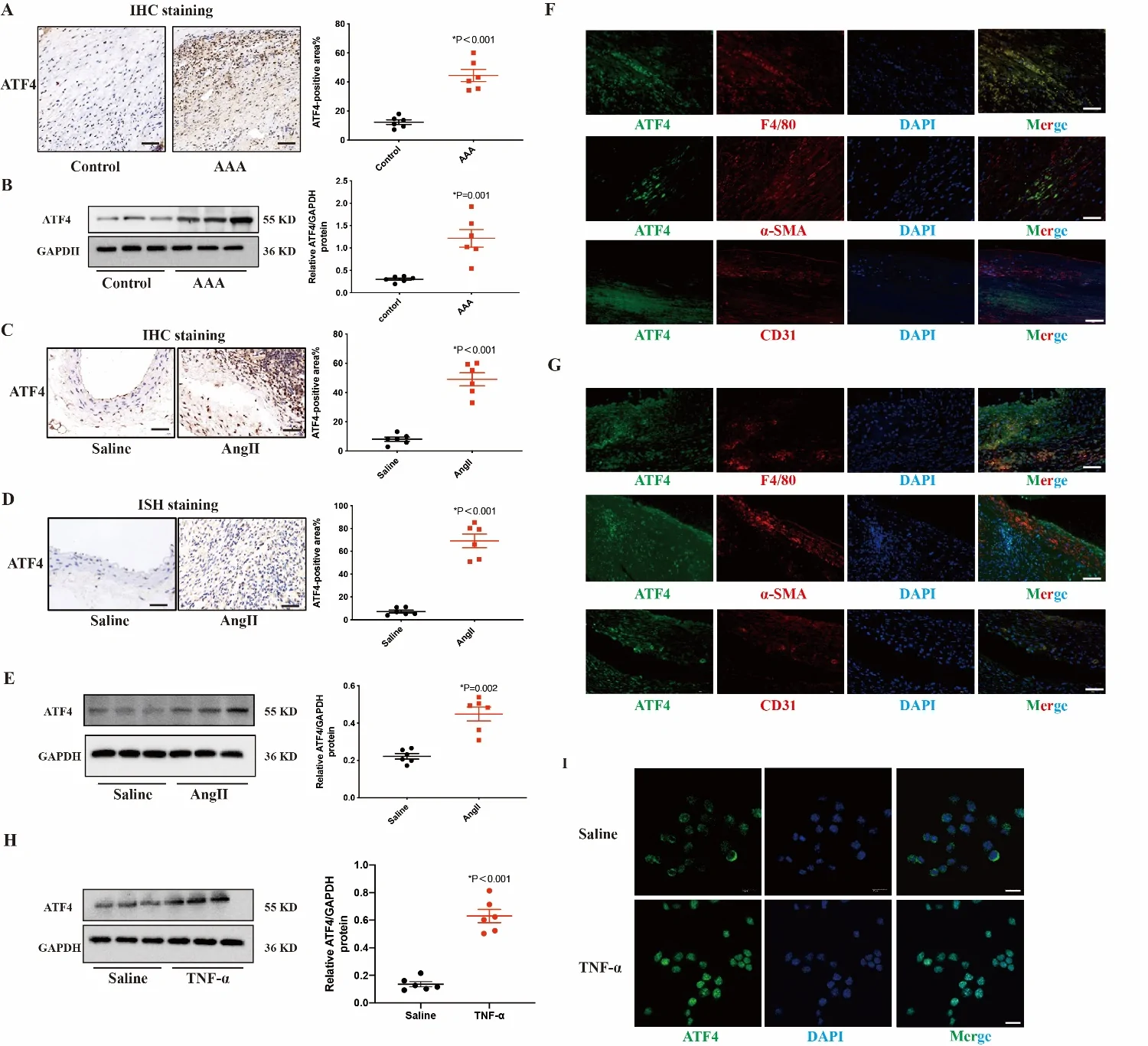
**Activating Transcription Factor 4 (ATF4) expression is significantly upregulated in human AAA and AngII-induced AAA of ApoE^-/-^ mice**. (**A**) Immunohistochemical results of ATF4 in human AAA tissue and control tissues. N=6 per group. (**B**) Western blot results of ATF4 in in human AAA tissue and control tissues. N=6 per group. (**C**) Immunohistochemical results of ATF4 in arterial tissues of saline and AngII-infused AAA in ApoE^-/-^ mice. N=6 per group. (**D**) In situ hybridization staining results of ATF4 in arterial tissues of saline and AngII-infused AAA in ApoE^-/-^ mice. N=6 per group. (**E**) Western blot results of ATF4 in arterial tissues of saline and AngII-infused ApoE^-/-^ mice. N=6 per group. (**F**) Immunofluorescence analysis of ATF4 (green), F4/80, ααSMA and CD31 (red) and DAPI in human AAA tissues. N=6 per group. (**G**) Immunofluorescence analysis of ATF4 (green), F4/80, ααSMA and CD31 (red) and DAPI in AngII-infused AAA in ApoE^-/-^ mice. N = 6/group. Scale bars: 50 μm. (**H**) Western blot results of ATF4 in RAW 267.4 cells incubated with TNF-α (10 ng/mL) for 24 h. N=6 per group. Data are presented as mean ± SD. (**I**) Immunofluorescence results of TNF-α stimulated RAW264.7 cells exhibited enhanced nuclear translocation of ATF4. Scale bars: 10 μm. Data are presented as mean ± SD. P-values in panels A-E, H were determined by the unpaired Student t-test.

To evaluate the involvement of ATF4 in AAA, we examined ATF4 expression in human AAA aortic tissue. Immunohistochemistry and western blot analysis results showed that ATF4 protein expression was significantly higher in human AAA tissues than in adjacent normal aortic tissues (Fig. 1A-B). We further investigated ATF4 expression in AAA mice model. Immunohistochemistry and western blots revealed significantly higher levels of ATF4 in Ang II-induced AAA mice compared with controls (Fig. 1C and E). Consistent with these results, in situ hybridization staining confirmed that ATF4 mRNA expression was significantly higher in Ang II-induced AAA mice compared with control mice (Fig. 1D).

Immunofluorescence staining showed that ATF4 protein colocalized with macrophages in AAA of humans and mice (Fig. 1F and G). In vitro, western blot showed that ATF4 expression was upregulated in RAW264.7 after AngII stimulation (Fig. 1H). Immunofluorescence showed AngII increased ATF4 expression and nuclear translocation (Fig. 1I). All of these findings suggested that macrophage-derived ATF4 was associated with AAA development.

**Figure 2.**
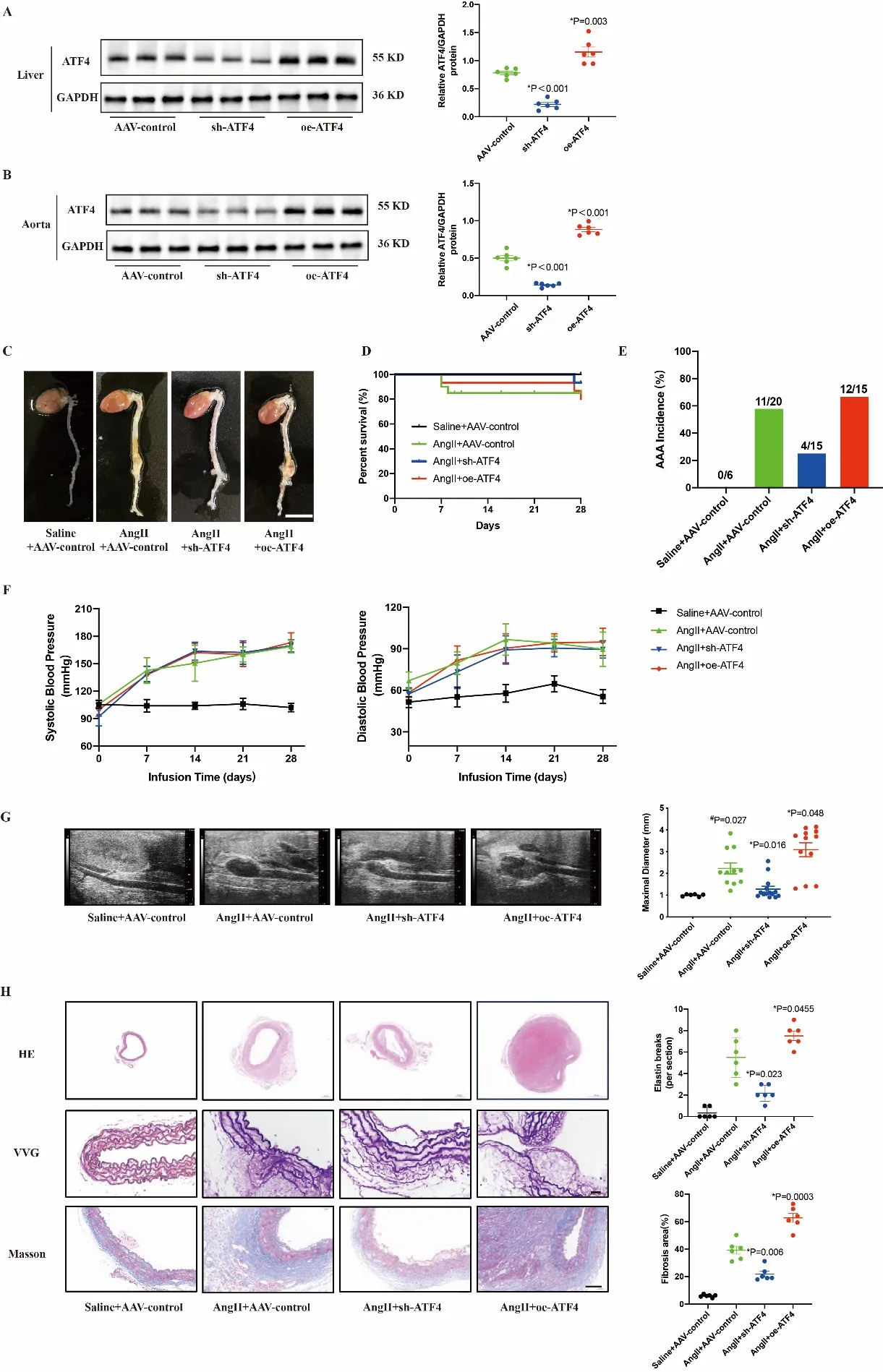
**ATF4 promotes AngII-induced AAA development in ApoE^-/-^mice**. (**A**) Western blot and analysis of liver tissues in ATF4-knockdown and ATF4-overexpression ApoE^-/-^ mice. N=6 per group. (**B**) Western blot and analysis of vessel tissues in ATF4-knockdown and ATF4-overexpression ApoE^-/-^ mice. N=6 per group. (**C**) Representative morphology of abdominal aortic specimens in 4 groups of mice: the Saline+AAV-control group, the AngII+control group, the AngII+sh-ATF4 group and the AngII+oe-ATF4 group. Scale bar: 1000 μm. (**D**)Survival curve showing the survival of mice at week 4 after Saline or AngII administration. N=6–20 per group. (**E**) Blood pressure of mice in 4 weeks after Saline or AngII administration. N=6–20 per group. (**F**) The incidence of AAA. N=6–20 per group. (**G**) Representative images from ultrasonography of abdominal aortas after AngII-infusion for 28 days, and quantification of maximal aortic diameter. N=6–14 per group. (**H**) Representative images and quantitative analysis of H&E staining, VVG staining and Masson’s Trichrome staining of abdominal aortas in mice. N=6 per group. Scale bars: 50 μm. Data are presented as means ± SD. Survival data in D were analyzed by the Kaplan-Meier method and compared using log-rank tests. P-values in panels G and H were determined by the two-way ANOVA after Bonferroni multiple comparisons.

### ATF4 promoted the development of AngII-induced AAA in mice

To explore the pathophysiological roles of ATF4 in AAA, we generated ATF4-knockdown and ATF4-overexpression mice. Genetic modifications were confirmed by western blot analysis of mouse liver tissue and aorta (Fig. 2A-B).

Four weeks after AngII infusion, the ATF4-knockdown (AngII+sh-ATF4) mice showed significantly smaller aneurysm formation compared with ApoE^-/-^ (AngII) mice, while ATF4-overexpression (AngII+oe-ATF4) mice showed aggravated AAA formation (Fig. 2C). KaplanαMeier curves showed that Ang II infusion led to the decreased survival compared with the Saline+AAV-control group, whereas ATF4 knockdown increased survival compared with the AngII+AAV-control group, and ATF4 overexpression had no effect on survival (100%, 75%, 93% and 80%, Fig. 2D). The incidence of AAA was 55%, 27% and 80% in the AngII+AAV-control, AngII+sh-ATF4 and AngII+oe-ATF4 groups, respectively, compared with that in the Saline group without AAA (Fig. 2E). Blood pressure was significantly elevated in mice at 28 days after the Ang II infusion mice compared to their baseline levels or Saline group. However, ATF4 knockdown and ATF4 overexpression had no effects on blood pressure (Fig. 2F). The maximal abdominal aortic diameter of ATF4-knockdown mice was significantly decreased whereas it was significantly increased in ATF4-overexpressing mice (Fig. 2G). The abdominal aortic dilatation, elastin degradation, and fibrosis were significantly higher in ATF4-overexpressing mice than in AngII+AAV-control mice, whereas abdominal aortic dilatation, elastin degradation, and fibrosis were significantly higher in ATF4-knockout mice than in AngII+AAV-control mice, whereas they were significantly higher in ATF4-overexpressing mice than in AngII+AAV-control mice. The aortic dilatation, elastin degradation, and fibrosis were significantly lower than those in the AngII+AAV-control group (Fig. 2H). These results suggest that ATF4 may play an important role in the deterioration of AAA.

### ATF4 aggravated inflammation, apoptosis, oxidative stress and MMP activity in AngII-induced AAA in ApoE^-/-^ mice

**Figure 3.**
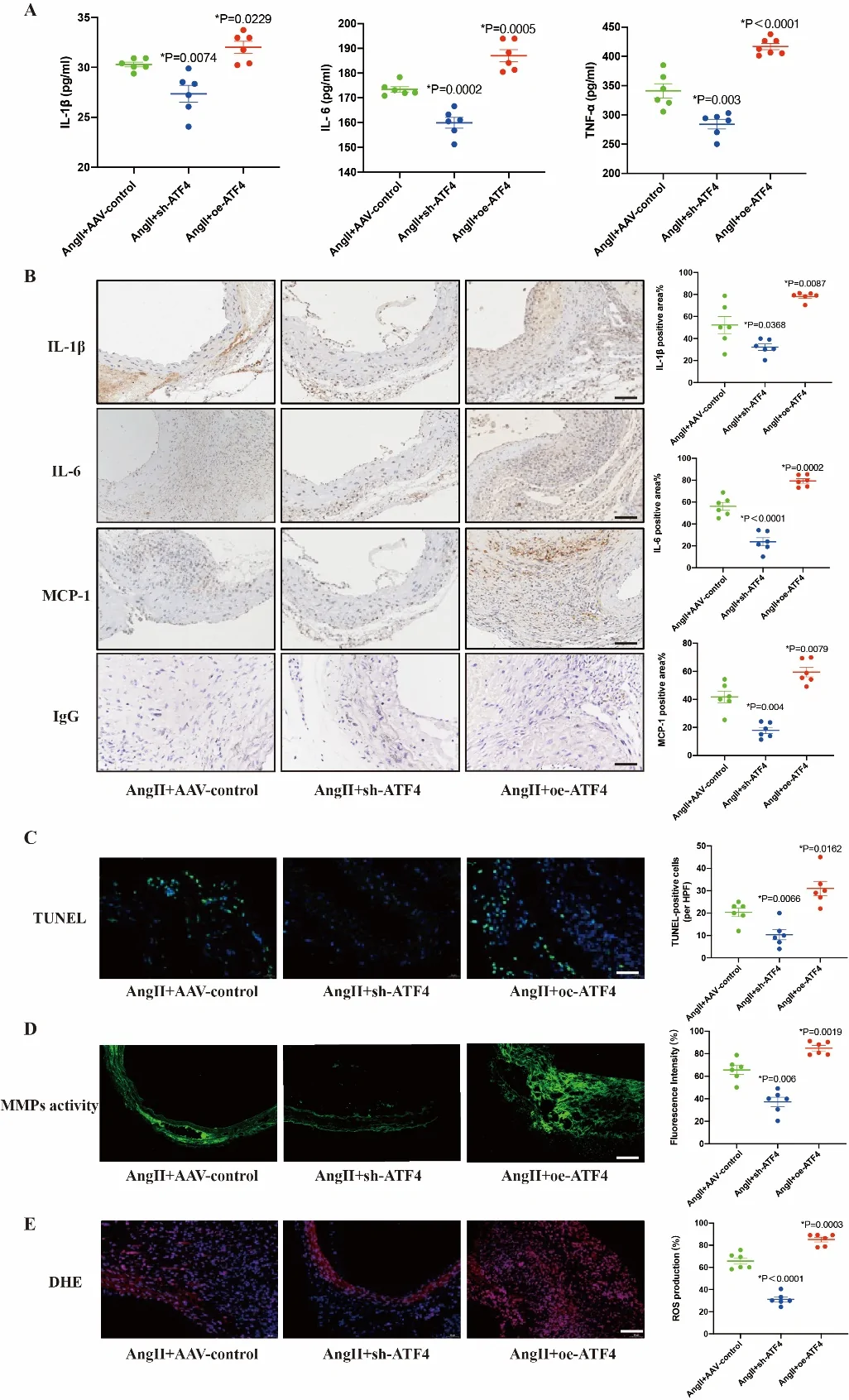
**ATF4 increased inflammation, apoptosis, MMPs activity and ROS levels in AAA mice**. (**A**) ELISA was used to test the plasma, IL-6, IL-1β and TNF-α in AngII-infusion mice group. N=6 per group.(B) Representative images and quantitative analysis of inflammatory factor IL-6, IL-1β and MCP-1 staining of abdominal aortas in each mice group. N=6 per group. Scale bars: 50 μm. (**C**) TUNEL staining of the abdominal aortas in AngII-infusion mice group. N=6 per group.Scale bars: 50 μm. (**D**) Representative photomicrographs and quantitative analysis of in situ zymography of abdominal aortas in each mice group. N=6 per group. Scale bars: 50 μm. (**E**) Representative photomicrographs and quantitative analysis of DHE staining of abdominal aortas in each mice group. N=6 per group.Scale bars: 50 μm. Data are presented as mean ± SD. P-values in panels A-E were determined by the two-way ANOVA after Bonferroni multiple comparisons.

As mentioned above, we found that ATF4 was mainly expressed in macrophages during AAA. Therefore, we measured plasma levels of pro-inflammatory factors such as IL-1β, IL-6 and TNF-α in AAA mice, and the results showed that the plasma levels of IL-1β, IL-6 and TNF-α were significantly increased in AngII+oe-ATF4 group compared with AngII+AAV-control group, while significantly decreased in AngII+sh-ATF4 group (Fig 3 A). Similarly, we observed that pro-inflammatory factors IL-1β, IL-6 and MCP-1 in AAA lesions were shown to be significantly increased in AngII+oe-ATF4 group and significantly decreased in AngII+sh-ATF4 group compared with AngII+AAV-control group by immunohistochemical staining (Fig. 3B).

Inflammation, apoptosis, oxidative stress and extracellular matrix degradation are regarded as the principal contributors to the development of AAA [2]. Therefore, we found that ATF4 knockdown reduced apoptosis assessed by TUNEL staining, and ATF4 overexpression aggravated apoptosis (Fig. 3C). ATF4 knockdown reduced oxidative stress assessed by DHE staining, and ATF4 overexpression aggravated oxidative stress (Fig. 3D). In-situ zymography assay revealed that MMPs activity was much higher in AngII+oe-ATF4 group than in AngII+AAV-control group and decreased in AngII+sh-ATF4 group (Fig. 3E). These results revealed that inflammation, apoptosis, oxidative stress and MMPs activity were significantly decreased in ATF4-knockdown mice. Conversely, the effect was aggravated by ATF4 overexpression.

### ATF4 promoted macrophages M1 polarization of AngII-induced AAA in ApoE^-/-^ mice

**Figure 4.**
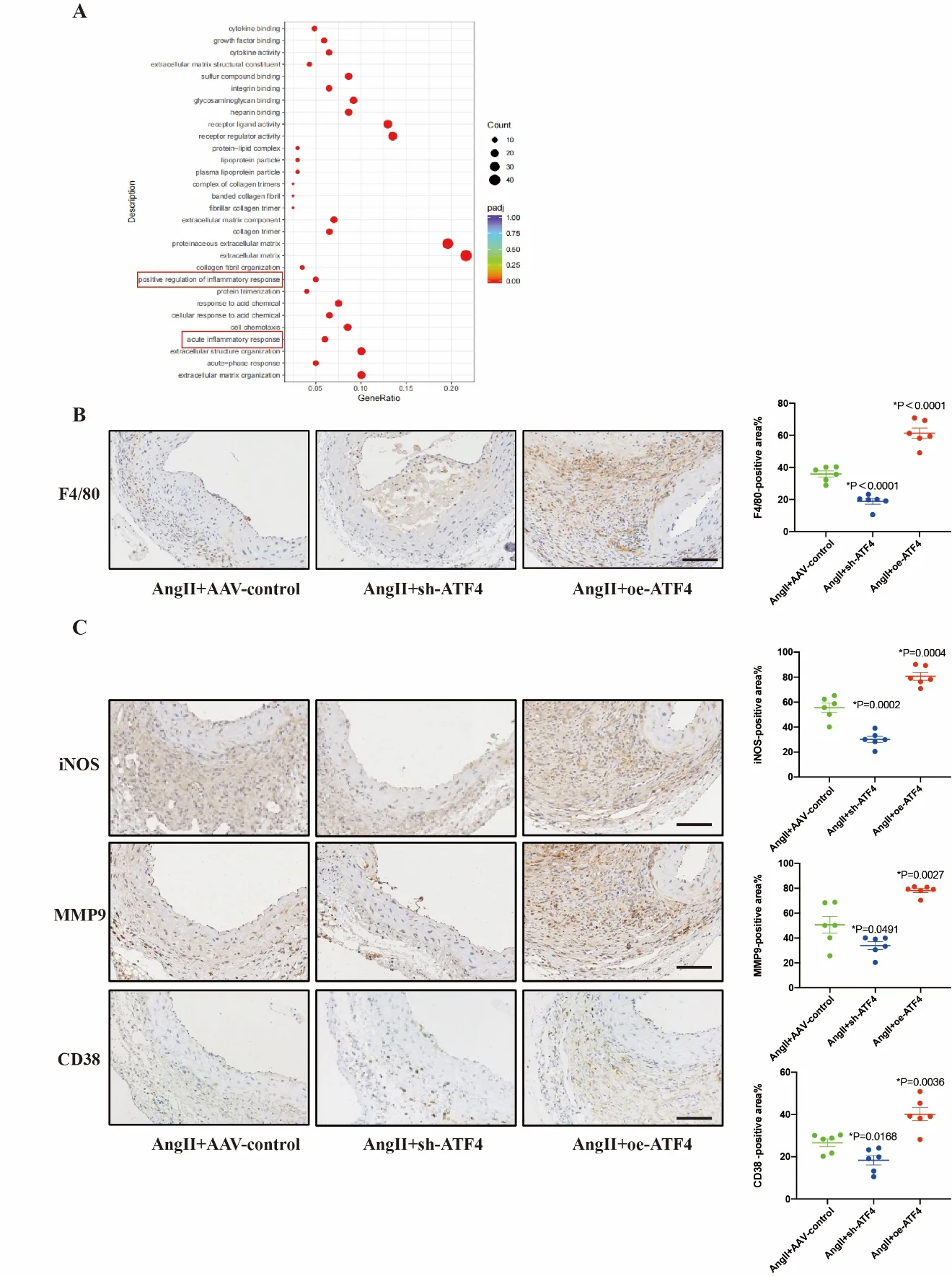
**ATF4 affects the inflammatory response and macrophage M1 polarization**. (**A**) Major biological process contributing to ATF4 function, as determined by gene ontology analysis of RNA sequencing results from vascular tissue of AAV-control and oe-ATF4 mice treated with an AngII pump for 28 days. The inflammatory response pathway is underlined in red. (**B**) Representative images and quantitative analysis of F4/80 staining of abdominal aortas in each mice group. (**C**) Representative images and quantitative analysis of iNOS, CD38 and MMP9 staining of abdominal aortas in each mice group. N=6 per group. Scale bars: 50 μm. Data are presented as mean ± SD. P-values in panels B and C were determined by the two-way ANOVA after Bonferroni multiple comparisons.

To acquire a clearer understanding into the mechanism of ATF4 in AAA, RNA-seq was conducted to detect its impact on transcriptomic profile. Gene ontology analysis showed that genes involved in acute inflammatory response were most significantly changed after ATF4 knockdown in AngII-treated ApoE^-/-^ mice (Fig. 4A). Macrophage activation and polarization are crucial for inflammation response [21]. Therefore, we analyzed the macrophage polarization among the three groups. Immunohistochemical analysis revealed that macrophage (F4/80) was increased in AngII+oe-ATF4 group compared to AngII+AAV-control group and decreased in AngII+sh-ATF4 group (Fig. 4B). Then, immunohistochemical analysis showed iNOS, CD38 and MMP9 in M1 macrophages in AAA lesions were significantly increased in AngII+oe-ATF4 group, while significantly decreased in AngII+sh-ATF4 group compared with AngII+AAV-control group (Fig. 4C).

**Figure 5.**
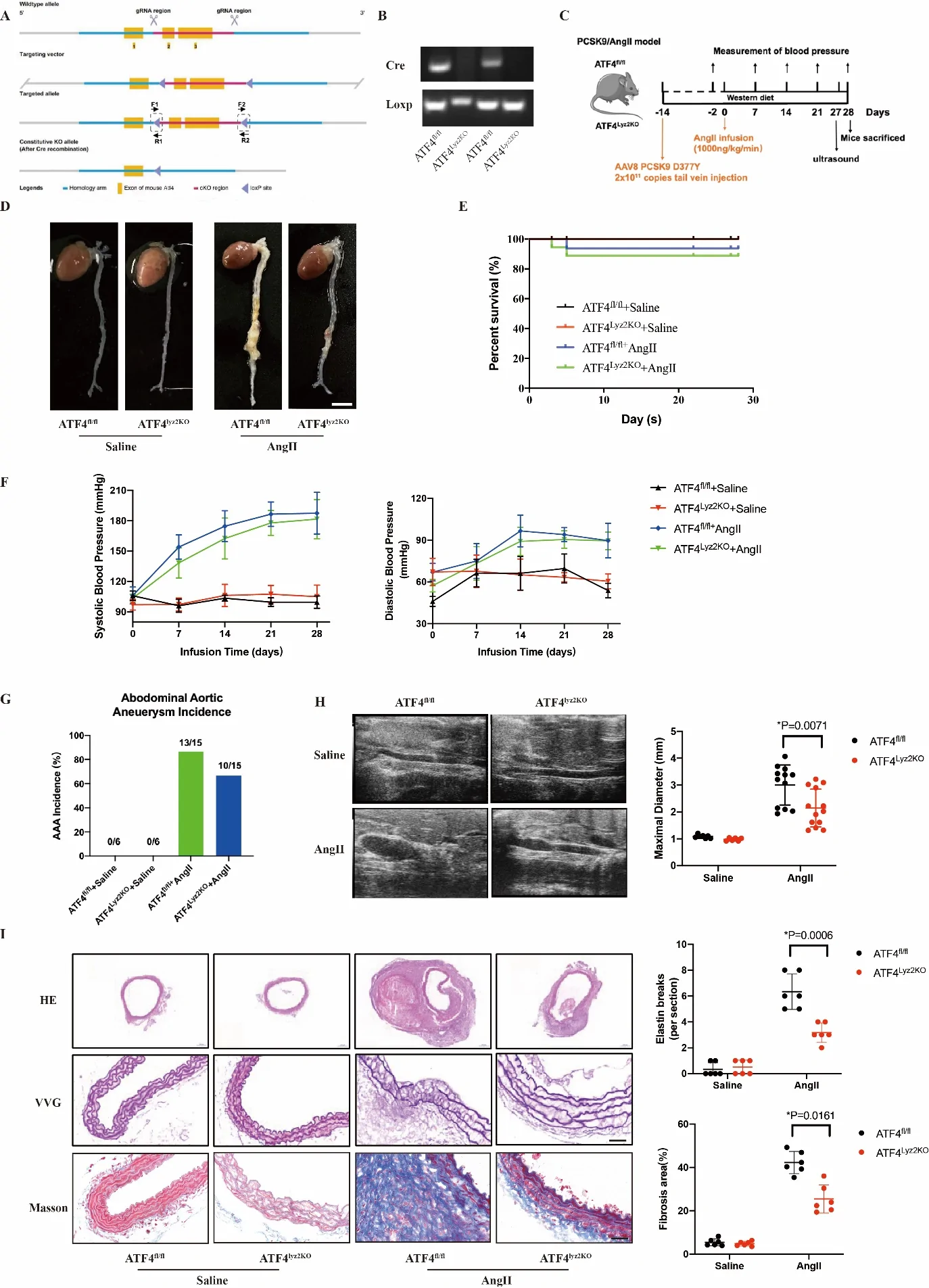
**ATF4 deletion in macrophages inhibits AAA development**. (**A**) ATF4^fl/fl^ mice were generated by inserting two Loxp sites between exon 2 and exon 3 of the mouse ATF4 gene. (**B**) The ATF4^Ly2KO^ transgene was confirmed by genotyping. Polymerase chain reaction of genomic DNA from ATF4^fl/fl^ and ATF4^Ly2KO^ mice was performed using specific primers. (**C**) Schematic protocol: 6-week-old ATF4 ^fl/fl^ and ATF4 ^Ly2KO^ mice were injected with AAV8 PCSK9, after 14 days saline or AngII infusion (subcutaneously) for 28 days. (**D**) Representative photographs of aortas from mice in the indicated groups. N=6 for Saline treatment; N=15 for AngII treatment. (**E**) Survival curve showing the survival of mice at week 4 after AngII administration. (**F**) Blood pressure of mice in 4 weeks after saline or AngII administration. (**G**) The incidence of AAA. (**H**) Representative images from ultrasonography of abdominal aortas after saline or AngII-infusion for 28 days, and quantification of maximal aortic diameter. N=6–13 per group. (**I**) Representative images and quantitative analysis of H&E staining, VVG staining and Masson’s Trichrome staining of abdominal aortas the indicated groups. N=6 per group. Scale bars: 50 μm. Data are presented as means ± SD. Survival data in E were analyzed by the Kaplan-Meier method and compared using log-rank tests. P-values in panels F-I were determined by the unpaired Student t-test.

### Macrophages specific deletion of ATF4 attenuated the development of AAA

To further investigate whether macrophages ATF4 was involved in the pathogenesis of AAA in vivo, we generated ATF4 macrophages specific knockout mice (ATF4^Lyz2KO^). Exons 2-3 were selected as the conditional knockout region. Two Loxp sites were inserted between exon 2 and exon 3 of the mouse ATF4 gene to generate ATF4^fl/fl^ mice, which were crossed with Lyz2-Cre mice to obtain ATF4^Lyz2KO^ mice (Fig. 5A). Genotypes were identified by PCR of tail tips (Fig. 5B). Next, 8-week-old male ATF4^Lyz2KO^ and their littermate ATF4^fl/fl^ mice were subjected to a single intravenous injection of AAV expressing a gain-of-function mutation of mouse PCSK9 (mPCSK9 D377Y) and fed a western diet, followed by AngII infusion to induce AAA (Fig. 5C).

**Figure 6.**
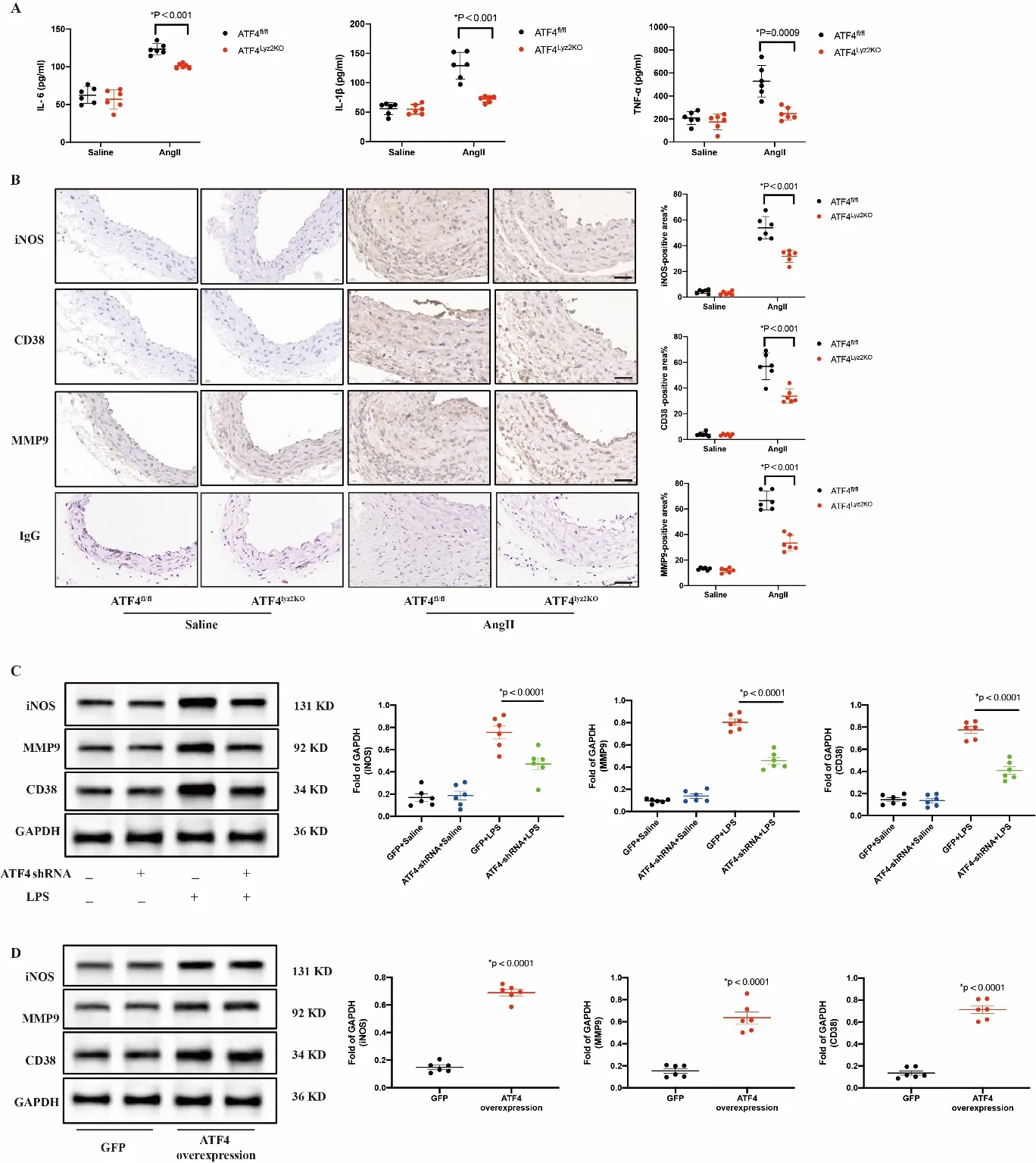
**ATF4 deletion in macrophages attenuate M1 polarization of macrophages**. (**A**) ELISA was used to test the plasma, IL-6, IL-1β and TNF-α in each mice group. N=6 per group. Data are presented as means ± SD. (**B**) Representative images and quantitative analysis of iNOS, CD38 and MMP9 staining of abdominal aortas in each mice group. N=6 per group. Scale bars: 50 μm. Data are presented as mean ± SD. (**C**) Western blot and quantitative analyses of iNOS, CD38 and MMP9 protein expression in RAW264.7 cells transfected with a GFP and ATF4-shRNA and treated with saline or 100 ng/mL LPS for 24 h. N=6 per group. Data are presented as mean ± SD. (**D**) Western blot and quantitative analyses of iNOS, CD38 and MMP9 of RAW264.7 transfected with the GFP and ATF4-overexpression lentiviral. N=6 per group. Data are presented as mean ± SD. P-values in panels A-D were determined by the unpaired Student t-test.

After 4 weeks of AngII infusion, the ATF4^Lyz2KO^ mice showed significantly smaller aneurysm formation compared with ATF4^fl/fl^ mice (Fig. 5D). There was no significant difference in survival rate and systolic blood pressure of mice between the two groups of mice after AngII infusion (Fig. 5E-F). Although saline-treated ATF4^Lyz2KO^ mice showed similar histology of aorta with ATF4^fl/fl^ mice, macrophages-specific ATF4 deficiency markedly decreased the incidence of AAA and the maximal diameters of the suprarenal abdominal aorta compared to those in ATF4^fl/fl^ mice after AngII infusion (Fig. 5G-H). In adddition, dilation of abdominal aorta, elastin degradation and fibrosis were accordingly reduced by ATF4 deficiency in macrophages (Fig. 5I). Serum levels of IL-6, IL-1β and TNF-α were significantly decreased in ATF4^Lyz2KO^ group compared with the ATF4^fl/fl^ group after treatment with AngII (Fig. 6A). Consistent with the ELISA results, western blot and immunohistochemical assay showed that iNOS, CD38 and MMP9 were significantly reduced in ATF4^Lyz2KO^ group compared with the ATF4^fl/fl^ group after treatment with AngII (Fig. 6B).

### ATF4 promoted M1 polarization by regulating expression of SMPD3 target genes

**Figure 7.**
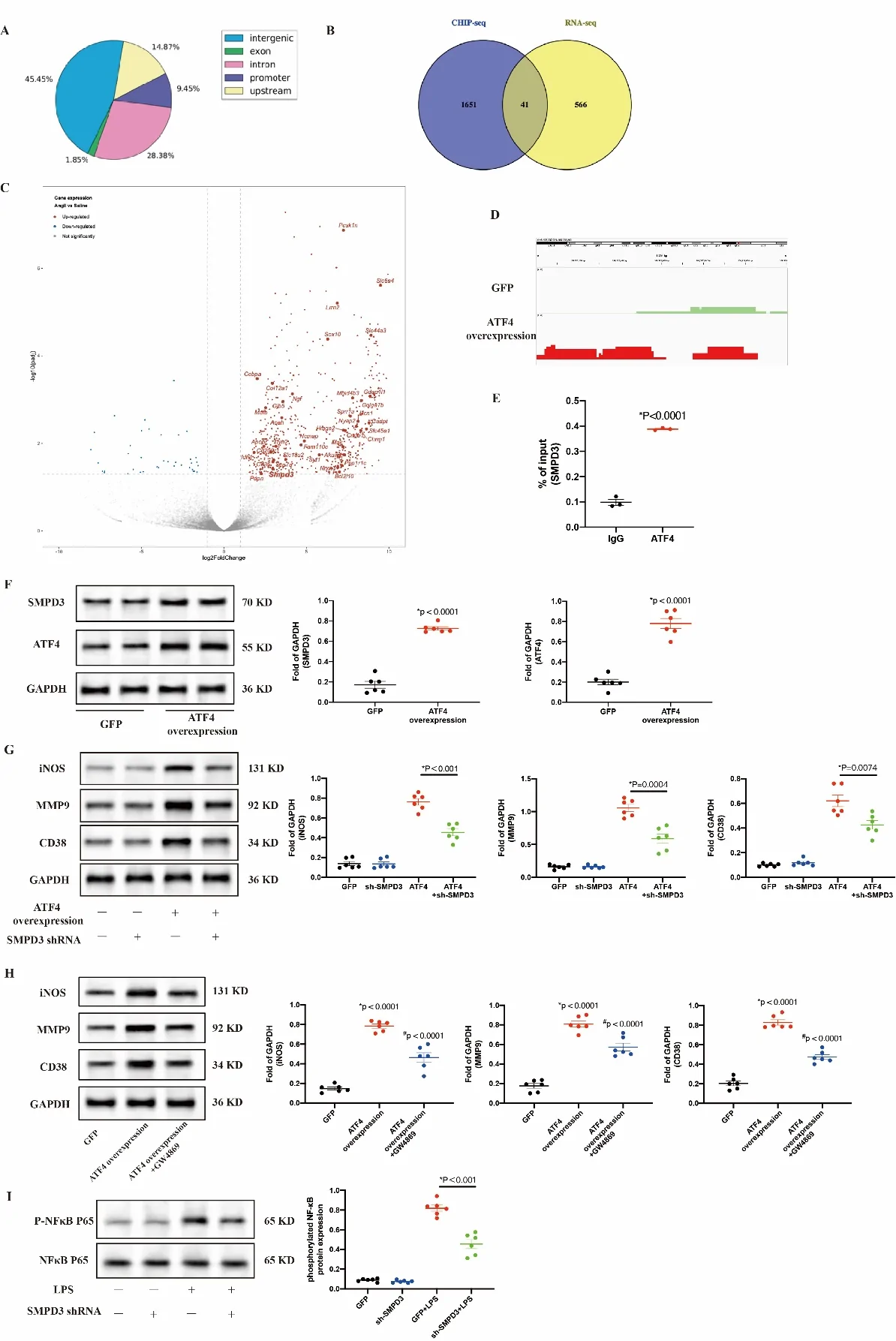
**ATF4 promotes M1 polarization by regulating expression of SMPD3 target genes**. (**A**) CHIP-seq analysis was performed in RAW264.7 infected with GFP or ATF4 overexpression with an ATF4 antibody. Chart demonstrates percent of peaks within each category. (**B**) ATF4-binded genes identified in the CHIP dataset (left circle) were overlaid upon genes exhibiting AngII-induced AAA of *ApoE^-/-^* mice expression in RNA-seq analysis (right circle) using Saline or AngII abdominal aortas. 41 genes were present in both datasets. (**C**) Volcano plot showing all genes in the RNA-seq analysis. (**D**)Map of the SMPD3 locus revealing ATF4 binding in RAW264.7 cells infected with GFP or ATF4 overexpression; aligned reads were visualized by Integrated Genomics Viewer 2.024. The signal of the GFP or ATF4 overexpression RAW264.7 cells is represented with green or red peaks, respectively. (**E**) The independent CHIP assay was performed upon GFP or ATF4 overexpression RAW264.7 cells to confirm ATF4 binding to the promoter regions of the SMPD3 gene. *P vs IgG Ab. Data are from 3 independent experiments. (**F**) Western blot and quantitative analyses of SMPD3 and ATF4 of RAW264.7 transfected with GFP and ATF4 overexpression; N = 6 per group. Data are presented as mean ± SD. (**G**) Western blot analysis of iNOS, MMP9, and CD38 in ATF4-overexpress RAW264.7 cells treated with the SMPD3 sh-RNA. (**H**) Western blot analysis of iNOS, MMP9, and CD38 in ATF4-overexpress RAW264.7 cells treated with the SMPD3 inhibitor GW4869. (**I**) Western blot analysis of p-NF-κB P65 and NF-κB P65 in RAW264.7 cells with GFP or SMPD3 knockdown treated with the PBS or LPS. N=6 per group. Data are presented as mean ± SD. P-values in panels E-I were determined by the unpaired Student t-test.

Transcription factor could exert biological activities by directly regulating the expression of target genes by binding to the gene promoter. To explore how ATF4 regulates M1 polarization in AAA, two strategies were utilized to identify downstream target genes regulated by ATF4 in macrophages. First, aortas were isolated from Saline or Ang II-infused ApoE-/- mice and subjected to transcriptome analysis. There were 566 differentially expressed genes (filtering criteria: P<0.01, fold change>1.5) between Saline and Ang II-infused ApoE-/- mice. Second, RAW264.7 was transfected with either ATF4-overexpression or GFP lentivirus. After upregulation of ATF4 expression was confirmed in the transfected cells (Fig. 7F), ATF4 CHIP-seq analysis was performed. We identified 1651 ATF4 target genes selectively enriched in ATF4-overexpressed cells. To narrow down the number of ATF4-mediated genes, we took the intersection of the Venn diagram; 41 common upregulated DEGs in both datasets were obtained (Fig. 7B). Volcano plots to identify the differential genes between ApoE-/- mice infused Saline or AngII (Fig. 7C). The intersection of two datasets was retained for further analysis, we found increased ATF4 binding at the SMPD3 promoter. CHIP-seq analysis revealed a significant ATF4-binding promoter region in the SMPD3 gene (Fig 7D). ChIP assay was performed to validate the binding of ATF4 to the SMPD3 promoter (Fig. 7E). SMPD3 had been identified to be associated with inflammation [22, 23].

To investigate whether ATF4 regulated macrophage polarization by SMPD3, we performed in vitro experiments. Western blot results showed SMPD3 expression significantly increased after upregulation of ATF4 expression in RAW264.7 (Fig. 7F). Knockdown of SMPD3 in RAW264.7 cells with SMPD3 shRNA significantly attenuated macrophage M1 polarization induced by ATF4 overexpression (Fig. 7G). Similarly, the SMPD3 inhibitor GW4896 significantly attenuated macrophage M1 polarization induced by ATF4 overexpression (Fig. 7H). It has previously been shown that the inhibition of SMPD3 attenuated TNF-α-induced phosphorylation of NF-κB [23]. Western blot results showed SMPD3 knockdown significantly reduced the LPS induced phosphorylation of the p65 subunit of NF-κB (Fig. 7I).

## DISCUSSION

**Figure 8.**
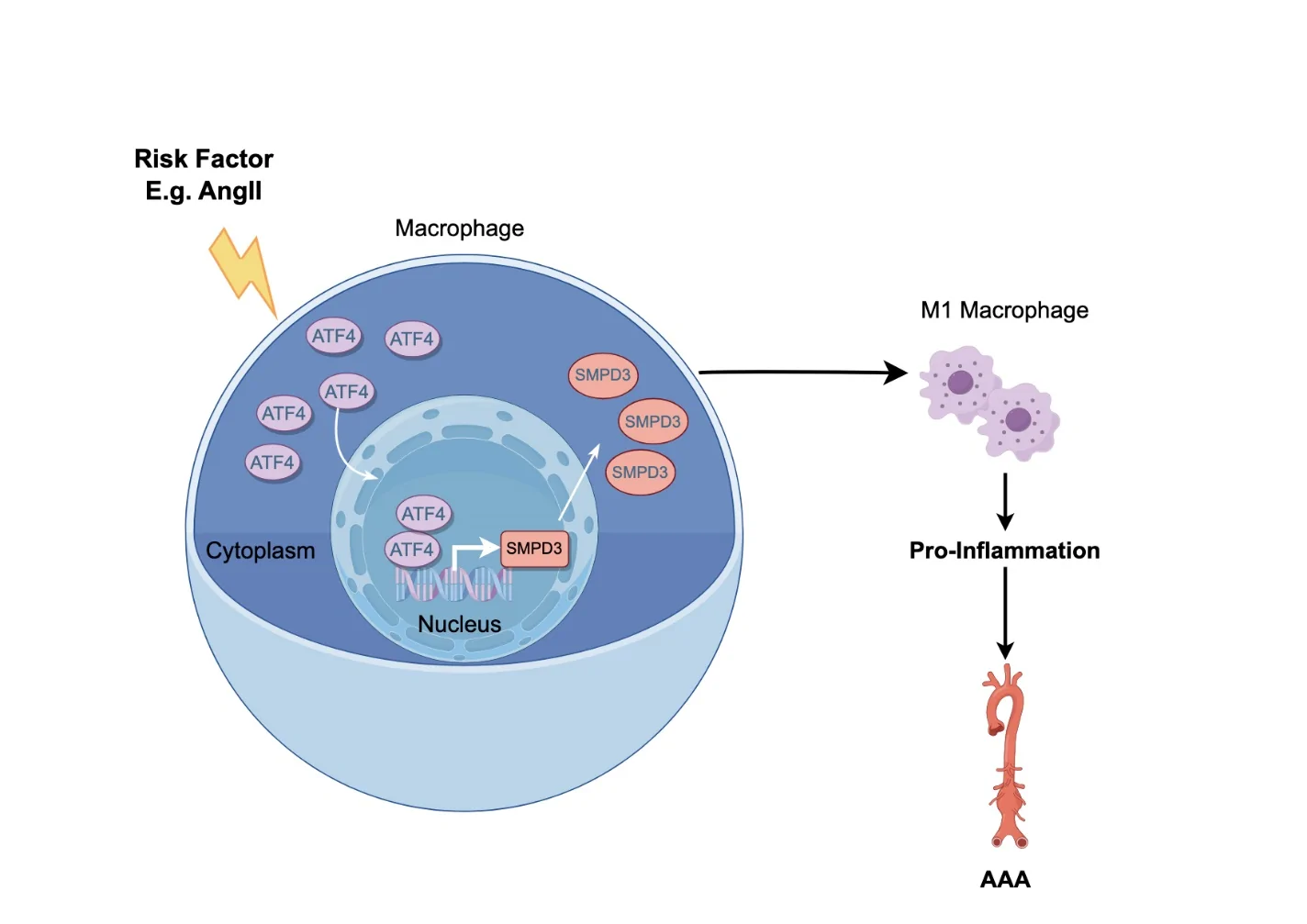
**Schematic illustrating the mechanism of ATF4 in AAA**. ATF4 activates SMPD3 transcription by binding it to its promoter in the nuclei of macrophages. ATF4 aggravates AAA mainly by upregulating SMPD3 contributing to inflammation response.

This study demonstrated for the first time a novel role for ATF4 in the pathogenesis of AAA (Fig. 8). Our results demonstrated that ATF4 is highly expressed in human AAA specimen as well as Ang II-induced lesions in a mouse model of AAA and co-localizes with the macrophages. Furthermore, ATF4 knockdown protected against AAA, while ATF4 overexpression resulted in a marked exacerbation of AAA, suggesting that ATF4 played an important role in AAA diseases. Macrophage-specific knockout of ATF4 effectively attenuated the progression of AAA via inhibiting macrophage M1 polarization. Mechanistically, we identified ATF4 as a key upstream transcription factor that regulated the expression of target genes SMPD3. ATF4 regulated macrophage M1 polarization through SMPD3.

AAA is considered as a chronic inflammatory vascular disease, as growing evidence suggests that a variety of inflammatory cells and factors play critical roles in the development of AAA [24]. Macrophage recruitment and M1 polarization contribute to the production of pro-inflammatory cytokine, and are early events in the progression of AAA [17]. Thus, blocking macrophage aggregation and M1 polarization may potentially reverse the vascular remodeling of AAA [25]. Macrophage polarization is regulated by several transcriptional factors [26]. The transcription factor interferon regulatory factor 5 (IRF5) is thought to be involved in the polarization of macrophage in response to inflammation and high expression is associated with M1 macrophage [27]. Our studies demonstrated that ATF4 aggravated the inflammatory response and promoted macrophage M1 polarization in mice with AAA. To further explore the role of ATF4 in macrophage of AAA, we constructed macrophage-specific ATF4 knockout mice using cre-loxP gene targeting. Our results suggested that macrophage-specific knockout of ATF4 attenuated the development of AAA. Macrophage-specific ATF4 knockout reduces macrophage M1 polarization and inflammation in AngII-induced AAA of mice. In RAW 264.7 cells, we found that overexpression of ATF4 polarized macrophage to M1.

ATF4 plays a crucial regulatory role in the inflammatory process, but there is no consensus on whether ATF4 promotes or inhibits inflammation. Recent studies have demonstrated that downregulation of ATF4 attenuates the endoplasmic reticulum stress-mediated neuroinflammation in mice model of Alzheimer's disease [28]. Downregulation of ATF4 ameliorated retinal inflammation in a mouse model of diabetes [29]. The ATF4-mediated histone deacetylase HDAC1 promotes the progression of acute pancreatitis [14]. Although most studies show that ATF4 has a pro-inflammatory effect, some studies also found that ATF4 has anti-inflammatory properties, which may be related to the disease models. A study indicated that the expression of ATF4 was decreased in inflamed intestinal mucosa of patients with active inflammatory bowel diseases, and overexpression of ATF4 down-regulated inflammatory factors including IL-1β and IL-6, while deprivation of ATF4 promotes intestinal inflammation [30]. In this study, we demonstrated that ATF4 aggravated inflammation and increased the expressions of pro-inflammatory factors IL-6, IL-1β, and MCP-1 in AngII-induced AAA of ApoE-/- mice.

Many transcription factors have been found to participate in aberrant genes modulation in AAA. Recent studies have shown that TCF7L1 (transcription factor 7-like 1) reduces the transcriptional activity of SRF and promotes the phenotypic transformation of VSMC, which aggravates the formation of abdominal aortic aneurysm [31]. In aortic dissection, activation of HIF-1α promoted the proinflammatory response and disruption of elastic fibers by increasing the expression of the HIF-1α target gene Adam17 [32]. To better understand the crucial role of ATF4 in AAA formation, we elucidated mechanism by using the CHIP-seq, RNA-seq and CHIP assay. we demonstrated that ATF4 interacted with SMPD3 target gene. As an important factor involved in macrophage polarization and inflammatory response, SMPD3 plays important roles in cardiovascular diseases, such as atherosclerosis [22, 23, 33], which is an important risk factor for AAA. Our results further revealed that ATF4 induced M1 polarization by regulating SMPD3 transcriptions through western blot analysis and rescue experiments. In RAW 264.7 cells, we found that overexpression of ATF4 polarized macrophage to M1. After SMPD3 inhibitor GW4869 treatment, the M1 macrophage polarization was reversed.

Current studies have shown the three classic methods for constructing AAA models are subcutaneous infusion of AngII, pancreatic elastase infusion and CaCl_2_ incubation [34]. In this study we choose AngII-induced AAA model, the most typical and generally accepted animal model of AAA, which could mimic most of the features of the human AAA [35]. In the part of the study to observe the role of macrophage-specific knockout ATF4 in AAA, we used PCSK9 in combination with AngII to induce the ATF4^flox/flox^ mice and the ATF4^LyZ2KO^ to establish AAA model. Tail vein injection of PCSK9-D377Y AAV8 and western diet feeding induced hyperlipidaemia in C57 mice mimiced the hyperlipidaemic features of ApoE^-/-^ [36].

Our study has several limitations. First, we used only full ATF4 knockout and overexpression mice to characterize the role of ATF4 in promoting AAA, rather than mice from the global knockout model, which could provide clearer evidence of causality. Second, we used only male mice because of the high success and reproducibility of this model. However, certain cardiovascular characteristics, such as blood pressure, serum lipid and vascular endothelial function, are different in mice of different sexes. Third, the relationship between SMPD3 and ATF4 could have been further examined in vivo model.

Taken together, our study provides evidence to support that ATF4 mediates macrophage M1 polarization by regulating the expression of target gene SMPD3, leading to the increase inflammatory responses, which further promotes the formation and development of AAA. These findings suggest ATF4 could be an emerging and potential therapeutic target for AAA.

## Data Availability

The data sets generated during and/or analysed during the current study are not publicly available for privacy reasons, but are available from the corresponding author on reasonable request.
